# Trend-following with better adaptation to large downside risks

**DOI:** 10.1371/journal.pone.0276322

**Published:** 2022-10-18

**Authors:** Teruko Takada, Takahiro Kitajima

**Affiliations:** 1 Graduate School of Business, Osaka Metropolitan University, Osaka, Japan; 2 Faculty of Commerce, Kumamoto Gakuen University, Kumamoto, Japan; DePaul University, UNITED STATES

## Abstract

Avoiding losses from long-term trend reversals is challenging, and trend-following is one of the few trading approaches to explore it. While trend-following is popular among investors and has gained increased attention in academia, the recent diminished profitability in equity markets casts doubt on its effectiveness. To clarify its cause and suggest remedies, we thoroughly examine the effect of market conditions and averaging window on recent profitability using four major stock indices in an out-of-sample experiment comparing trend-following rules (moving average and momentum) and a machine-classification-based non-trend-following rule. In addition to the significant advantage of trend-following rules in avoiding downside risks, we find a discrepancy in the optimum averaging window size between trend direction phases, which is exacerbated by a higher positive trend direction ratio. A higher positive trend direction ratio leads to poor performance relative to buy-and-hold returns. This discrepancy creates the dilemma of choosing which trend direction phase to emphasize. Incorporating machine-learning into trend-following is effective for alleviating this dilemma. We find that the profit-maximizing averaging window realizes the level that best balances the dilemma and suggest a simple guideline for selecting the optimum averaging window. We attribute the sluggishness of trend-following in recent equity markets to the insufficient chances of trend reversals rather than their loss of profitability. Our results contribute to improving the performance of trend following by mitigating the dilemma.

## 1 Introduction

Stock markets have recurring upward and downward trend direction phases, and investors can lose much of the earned profit during a maximum drawdown. However, avoiding losses from trend reversals is a challenging task involving fundamental difficulties. This study considers the problem of long-term trend direction classification to help avoid losses from such trend reversals.

The essential difficulty of the long-term trend direction classification problem lies in the distinction between noisy daily stock price fluctuations and the rare large price trend direction reversals. Given the tendency for large, sudden trend reversals in the stock market after a long, stable, and continued upward trend, we can interpret the difficulty for investors as a decision between “keep riding the upward long-term trend” or “be cautious about sudden trend reversals”.

This situation corresponds to the *stability-plasticity dilemma* [1] that both human and learning machines often encounter in the process of knowledge acquisition when confronted with new environments, typically involving the problem of non-stationarity (or concept drift) with class imbalance. Non-stationarity and class imbalance constitute a combination representing the most challenging current problems for computational intelligence studies [2]: the machine classification problem is complicated by non-stationarity, where the underlying data distribution is time-varying and insufficient information on the minority class complicates it further.

In particular, dealing with non-stationarity is highly challenging [3]. Several studies propose remedies to address non-stationarity by classifying the types of non-stationarity. However, they consist mainly of general-purpose theory-oriented approaches [4–6] are critical of their practical performance. They emphasize the importance of simplicity in the model by tailoring it to each specific application problem. Because of such difficulties dealing with non-stationarity, trend direction classification studies using machine classification focus on the short term, with temporal horizons of daily and monthly at most.

The handling of simultaneous concept drift and class imbalance is attracting increased attention, especially in real-world online learning. However, it is still an emerging research area, and very few studies address the simultaneous occurrence of these problems [7]. Applying the proposed solutions to real-world data is often difficult because the expected characteristics of the data are usually unknown [8].

As a trading strategy, *trend-following* is possibly the only approach that addresses the *long-term* trend direction classification problem while providing the desired simplicity. Trend-following is a form of technical analysis that aims to ride an upward trend formation while avoiding losses by identifying the downward trend reversals in time. Trend-following rules generate buy (sell) signals when users identify the start (end) of a trend formation. In the simplest formulation, a single parameter, the *averaging window*, also called the *look-back period*, balances “the stability of the long-term trend” and the “sensitivity (plasticity) to trend direction changes”. That is, the dilemma in choosing to emphasize the “clear capture of an upward trend” and “sensitivity to trend reversal signals”. Hence, selecting the appropriate averaging window becomes the key to improving trading performance.

This study aims to empirically clarify the source of the recent unstable and diminished returns of trend-following in equity markets and suggest remedies to realize their improved advantage in avoiding long-term trend reversals. We conduct the following two studies. First, we perform a comprehensive out-of-sample comparative analysis that focuses on the effect of trend direction and strength on profitability in relation to the averaging window size. We also examine the effect of the imbalance degree of the trend direction ratio. We compare the simplest form of standard trend-following rules: time-series momentum (TMOM) and single moving average cross over (MACO), and the ordinal support vector machine (OSVM), a simple machine-classification-based non-trend-following rule. To minimize the risk of data snooping, we analyze data from major daily stock indices (the NYSE, NASDAQ, FTESEALL, and TOPIX) for the sample period of 1973 to 2019. The out-of-sample test period is from 1995 to 2019, when researchers began to report unstable and reduced profitability, especially in the U.S. markets.

Second, we introduce a machine-classification-based trend-following rule, the long-term trend direction classifier (LTDC). We adopt this approach to contrast the features specific to trend-following rules and examine the effectiveness of incorporating simple machine-classification-based remedies in varying market conditions involving the problem of non-stationarity and the class imbalance. With only the target price series, the LTDC learns the *stable* long-term MA-smoothed trend direction via a support vector machine (SVM) classifier in relation to the trend reversal signs *sensitively* detected via short-term daily return moments. The difference between OSVM and LTDC is the target to learn: OSVM learns the *raw* short-term (e.g., monthly) stock price change direction, but the LTDC learns the MA *smoothed* long-term stock price trend direction that realizes stable learning. We set the other conditions the same: Both OSVM and LTDC employ SVM which is advantageous for varying market conditions compared to many classifiers, and contain remedies for an imbalanced direction class.

This study offers two contributions to the literature. First, the results empirically illustrate the underlying causes of the recent unstable performance, which, as the study demonstrates, does not imply the loss of profitability. In addition to the significant advantage of trend-following rules in avoiding downside risks, we find a discrepancy in the optimum averaging window size between positive and negative trend phases, which is widened by higher positive trend direction ratio, reflecting the degree of discrepancy. A higher positive trend direction ratio leads to poorer performance relative to buy-and-hold returns (BHR), and the effect exceeds that of volatility. That is, the discrepancy creates a dilemma regarding which trend direction to emphasize when selecting the main control parameter, the averaging window, resulting in the unstable performance of trend-following due to inappropriate averaging window size to adapt to the market conditions black.

Second, we provide effective suggestions to mitigate the dilemma of averaging window selection by focusing on the essential difficulty of trend-following rules. We demonstrate the effectiveness of incorporating the simplest possible machine classification-based remedies into standard trend-following rules to alleviate the negative effects of non-stationarity with class imbalance on performance. We validate the advantages of trend-following rules in protecting against long-term trend reversals via bootstrap significance tests. Among the compared rules, the LTDC machine-classification-based trend-following rule has the highest returns and robustness most of the time. Particularly, we extend the advantage of downside protection of trend-following to markets with higher positive direction ratios. Moreover, we suggest a simple data-based guideline to select the optimum averaging window: the mean return level of the target price series as a practical indicator reflecting both the key information of the trend direction ratio and the trend strength. This helps to attain profit closer to the maximum achievable level.

Our results confirm that trend-following rules demonstrate their true value in markets with sufficient opportunities for trend reversals. Their protection ability relative to BHR is not fairly rewarded during a stable, upward trend period. This explains why the profitability of trend-following rules appears more commonly in commodity markets than in stock markets. Our results also suggest that the recent reduced profitability of trend-following rules relative to BHR in U.S. equity markets could be explained by the insufficient downside risks; that is, it does not outperform BHR due to the prolonged upward trend periods in the dataset. We extend the advantage of trend-following rules to markets with higher positive trend direction ratios by providing effective remedies to mitigate the dilemma of which trend direction phase to emphasize in averaging window selection. These remedies are commonly applicable for all trend-following rules with any machine classifier, enabling further enhancement of the downside risk protection ability of the trend-following rules.

The remainder of the paper is organized as follows. Section 2 summarizes the related studies. Section 3 explains the trading rules compared including the proposed LTDC method and remedies. Section 4 describes the data, while Section 5 outlines the research design. Section 6 presents the results of a comprehensive comparative out-of-sample experiment. Section 7 provides discussion on the remaining problems, and section 8 concludes.

## 2 Related studies

### 2.1 Trend-following studies in finance

Trend-following strategies are widely used among practitioners and are receiving growing scholarly attention because of their potential for high profitability and because they outperform BHR. See, for example [9–14]. Above all, trend-following strategies are as strong during market stress periods with downside risk or high volatility phases [13, 15, 16]. In particular, prior studies document the profitability of trend-following rules in volatile assets [17–19].

However, the recent reduced or unstable profitability of trend-following rules in equity markets has become a subject of discussion, casting doubt on their effectiveness. In U.S. equity markets, particularly for indices and large stocks, some studies report diminished or unstable profitability from trend-following rules since around 1990 [13, 20–22]. Others report a similar tendency in the equity markets of other developed countries [23] and foreign exchange markets [24], though no studies report evidence of a decrease in profitability in commodity futures markets [25].

Empirical research suggests several factors leading to diminished or unstable profitability from trend-following rules in U.S. equity markets. According to [26], the decrease in several anomalies, including the momentum effect, is attributed to increased liquidity and trading activity. The study about the out-of-sample and post-publication return predictability by [27] suggests data mining effects, as well as the possibility that traders learn about mispricing from academic publications.

Focusing on the fundamental problems of non-stationarity, a few studies provide theoretical evidence that the unstable profitability of trend-following rules is caused by the difficulty of keeping the trading model optimal, where the averaging window size and trend strength are the key factors. Assuming an autoregressive return process, [28] study the relationship between averaging window size and the trading performance of MA rules theoretically, and find that the dominant factor affecting trading performance changes from autocorrelation to the drift around a few months’ look-back period. Moreover, their in-sample study demonstrates that the drift (autocorrelation) phase is characterized by an increase (decrease) in the Sharpe ratio as a function of the averaging window size.

Authors in [29] theoretically compare and contrast TMOM and MA rules to summarize important properties of trend-following rules. Assuming an autoregressive return process, they demonstrate a theoretical significant positive effect of trend strength on the similarity in forecasting accuracy between the trading rules of TMOM and MA. Given the difficulty of identifying trend strength and the time-varying optimal averaging window size, they conclude that maintaining the optimum averaging window is difficult, and that the robustness to the changes in the averaging window size determines whether MA outperforms TMOM in terms of profitability. However, the overall picture of how trend strength and averaging window affect the profitability behavior of trend-following is not yet empirically clarified.

### 2.2 Measures for non-stationarity with class imbalance in machine learning

Whereas non-stationarity with class imbalance has become a critical issue in applying machine learning to real world problems, the combined problem is addressed by very little work [5, 7]. There are several measures developed to overcome non-stationarity without class imbalance problem, which can be classified into the following three categories: 1) adaptive base learners, 2) adaptive training set formation, and 3) ensemble techniques [4, 5]. The second category modifies the training set for better adaptation, which we can further decompose into windowing techniques and weighting techniques [5]. For the purpose of realizing practical performance, however, [4–6] criticize the application of general-purpose theory-oriented remedies, and emphasize the importance of designing the simple adaptation algorithm customized to the specific nature of the non-stationarity in each application problem.

This study focuses on the windowing techniques in the second category because 1) it is the simplest and 2) the standard trend-following trading rules are classified as windowing method in that it modifies the target trend direction whose scale is controlled by averaging window or look back period. Windowing techniques select an appropriate set of previous data to train a classifier. How to use the past data streams depends on the type of non-stationarity the user confronts, the nature of available data, and required computational costs. The most important drawback of the windowing techniques is that it is impossible to determine the appropriate window size for any given problem in advance [5]. Moreover, the user confronts a stability-plasticity dilemma in optimizing window size between small window (fast adaptivity) and large window (better generalization) [30].

The remedies developed for class imbalance under stationarity are classified as: 1) resampling techniques, 2) algorithm-level methods, 3) ensemble learning. However, they are not effective for non-stationarity data streams because they cannot handle drifts that affect classification boundaries [7]. Employing evaluation measures giving more weight to the minority class is simple and easily applicable, and reported as effective to handle imbalanced data stream [31, 32]. Whereas there are several proposed measures such as G-mean, recall, and the Kappa statistic, for example, Kappa statistic is reported as advantageous relative to the others for real-time unbalanced class measure of accuracy [8, 33].

## 3 Trading rules and remedies

### 3.1 Standard trend-following rules

#### 3.1.1 Time series momentum rule

Let *t* be the end date of the most recent month. The observed series of raw daily closing prices at date *t* are *P* = {*P*_1_, *P*_2_, …, *P*_*t*_}. The trend-following rules make trading decisions based on the direction of the price trend during the past *d*_*L*_ days up to date *t*, where *d*_*L*_ is the *look-back period* or *averaging window*. The difference between the MA and TMOM is how we obtain the price trend. For both rules, the time scale of the long-term trend changes as *d*_*L*_ changes.

TMOM introduced by [11] differs from the conventional cross-sectional relative returns in that it focuses purely on a security’s own past returns. The TMOM rule generates a buy (sell) signal at date *t* if the past *d*_*L*_-day return is positive (non-positive). That is, the TMOM generates a buy signal at date *t* if

$$\begin{array}{c} TMOM_{t,d_{L}}=P_{t}-P_{t-d_{L}}>0, \end{array}$$ (1)

and a sell signal when $TMOM_{t,d_{L}}\leq 0$. The source of the conventional cross-sectional momentum profits originates mostly from the time-series dependence in realized returns rather than the cross-sectional factors according to studies of momentum profit decomposition by [34, 35]. A 12-month look-back period and a one-month holding period is reported to generate the highest profitability for all securities [11].

#### 3.1.2 Moving average-based rule

MA-based trading rules have many variations, and we use the most popular formulation, MACO. Using data on 28 commodity futures markets, [10] demonstrate the superior performance of dual MA crossovers and channel strategies relative to momentum strategies. They emphasize that employing the typical research design for momentum strategies using intermediate investment horizons is the key for a profitable result using MA strategies. In addition, they document that weakly significant individual results can be significantly powerful when they are aggregated across markets. We obtain the long-term smoothed price at date *t*, *y*_*t*_, by the *d*_*L*_-day simple MA, formulated as

$$\begin{array}{c} y_{t}=\frac{1}{d_{L}}\sum_{i=0}^{d_{L}-1}P_{t-i}. \end{array}$$ (2)

Here, the averaging window, *d*_*L*_, controls the degree of smoothing. Let *z*_*t*_ be the short-term MA with an averaging window of *d*_*s*_, where *d*_*s*_ < *d*_*L*_. The MACO generates a buy signal on date *t* when

$$\begin{array}{c} MACO_{t,d_{L},d_{s}}=z_{t}-y_{t}=\frac{1}{d_{s}}\sum_{i=0}^{d_{s}-1}P_{t-i}-\frac{1}{d_{L}}\sum_{i=0}^{d_{L}-1}P_{t-i}>0, \end{array}$$ (3)

and a sell signal when $MACO_{t,d_{L},d_{s}}\leq 0$. In the simplest form, we use the price at time *t*, *P*_*t*_, as the short-term MA, *z*_*t*_.

### 3.2 Long-term trend direction classification rule

#### 3.2.1 Overview of the method

LTDC is a machine-classification-based trend-following rule which is designed as simplest possible to realize effective handling of non-stationarity with class imbalance that investors encounter to avoid losses from large trend reversals. The LTDC trading rule predicts future long-term trend direction by stably learning the past relationship between the MA-smoothed long-term trend direction and sensitively capturing the varying market conditions expressed as the recent return moments using the SVM classifier.

**Fig 1:**
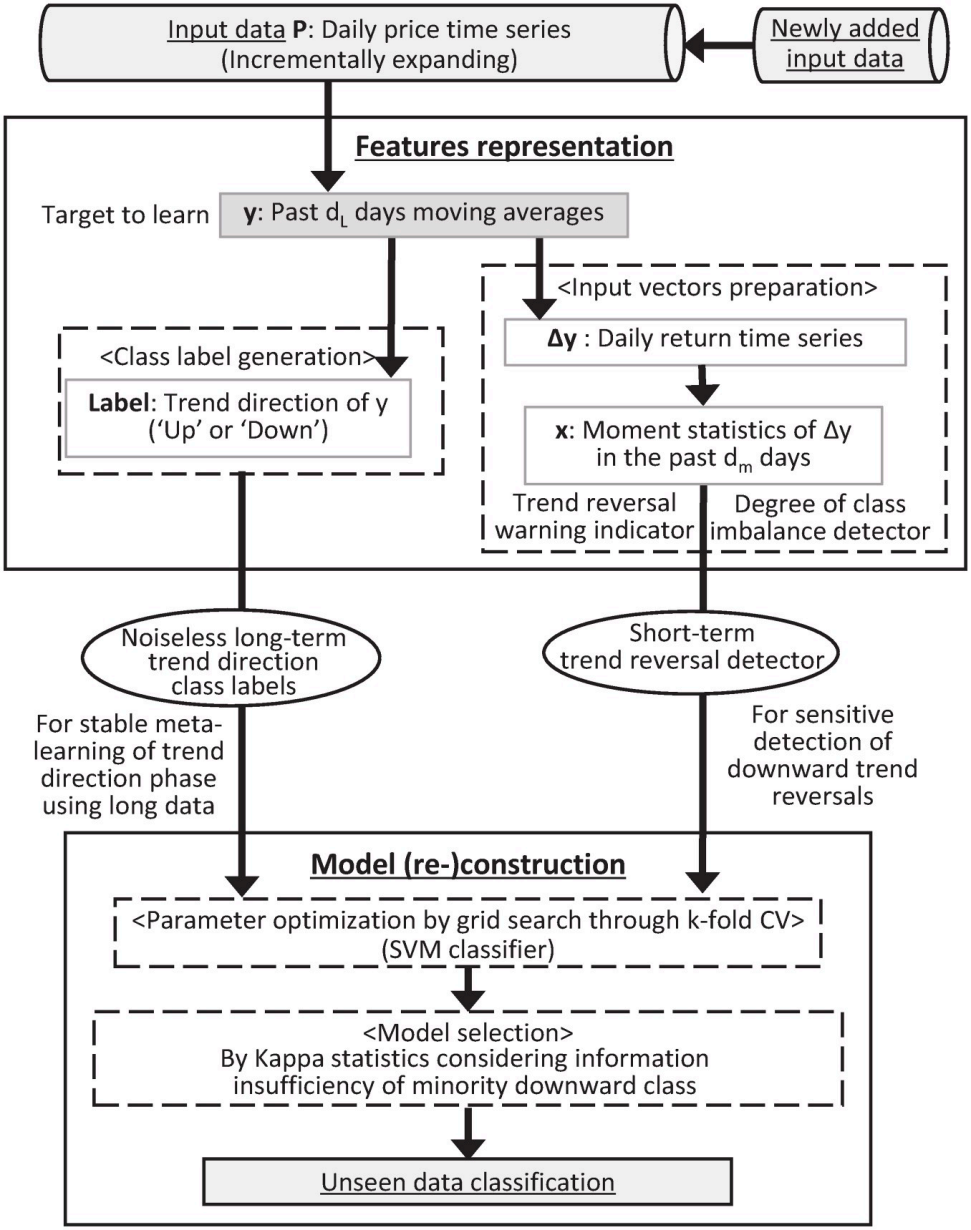
Schematic layout of the long-term trend direction classification method.

Fig 1 shows the overall architecture of the LTDC system. The system consists of two steps before the unseen data classification: feature representation and classification model (re-)construction. Given raw daily price time-series *P* and averaging window *d*_*L*_, the feature representation step prepares the class labels of *y* (*d*_*L*_-day MA of *P*). In the next step, Δ*y* is computed as daily return time series of *y* during past *d*_*m*_ days, and the obtained moment statistics of Δ*y* is used as input vectors **x**. Here, the feature is that class labels are generated not from the *raw* price series *P* but from the MA *smoothed* price series *y* representing the long-term trend movement. We directly monitor the changes in return density shape (i.e., market conditions) through the moment statistics of the short-term daily smoothed price change series. Any machine classifier can be used in our framework, but we select binary SVM classification for its robustness under concept drift. Parameter optimization is by grid search through k-fold CV using maximum training data period to keep information of rare large trend reversals, which reduces the need of frequent model re-construction. We select the model based on Kappa statistics as they provide the sensitivity to detect a minor downward trend direction. Provided the input vectors **x** (recent return moments describing market condition), SVM classifies the corresponding direction of target trend *y*. LTDC generates a buy (sell) signal if the classified direction is positive (negative).

We provide a detailed explanation of the respective parts and the implementation below.

#### 3.2.2 Support vector machine classification

The SVM was developed by [36] and it has since been widely applied because of its excellent classification performance. Among various methods, we select SVM for machine classifier of LTDC because it is simple but realizes robust online learning under non-stationarity [4, 30]. Actually, the SVM is one of the most used classifiers in online concept drift learning studies [37]. In forecasting stock price direction, SVM is one of the best algorithms [38–41]. Specifically, it is reported as advantages in environments with non-stationarity and noise relative to competing methods [39, 42].

The SVM is based on the principle of structural risk minimization and statistical learning theory. The basic idea behind SVM is to map the original data points in an input space to a high-dimensional feature space using linear classification. The learned classification rule is then mapped back to the original input space. The standard model, C-support vector classification, is as follows. Consider the training dataset **x** composed of N input vectors **x**_1_, ⋯, **x**_*N*_ corresponding to target labels *y*_1_, ⋯, *y*_*N*_, where *y* ∈ {−1, + 1}. We set labels *y*_*i*_ to −1 (+1) if the trend direction is downward (upward). Let *ϕ*(**x**) denote a fixed feature space transformation for some weight vector **w** and bias parameter *b*. Then, the binary soft margin classification problem is

$$\begin{array}{c} \underset{w,b,\xi }{\text{arg}\;\text{min}}\;\frac{1}{2}{\left||w|\right|}^{2}+\frac{C}{N}\sum_{n=1}^{N}\xi_{i},\;\left(C>0\right),\;\text{subject}\;\text{to}\;y_{i}\left(w^{T}\phi \left(x\right)+b\right)\geq 1-\xi_{i}, \end{array}$$ (4)

where *ξ*_*i*_ ≥ 0, *i* = 1, ⋯, *N* is the slack variable tolerating misclassification and parameter *C* controls the trade-off between a large margin and misclassification errors. That is, a larger *C* increases the rate of recognition but reduces generalization performance through overfitting.

#### 3.2.3 The stable class label of the long-term trend direction

The LTDC target to classify is not the ordinal *raw* (typically daily) price change direction but the *smoothed* long-term price trend direction, which facilitates the learning task of distinguishing the large downward trend reversals from noisy fluctuations. Among the various methods for modifying the training set for better adaptation, the windowing technique is the most common approach and provides clearer probability estimation on unseen data [4, 5]. To obtain the long-term trend, we select MA smoothing as its performance is superior to that of the TMOM rule [13, 29].

**Fig 2:**
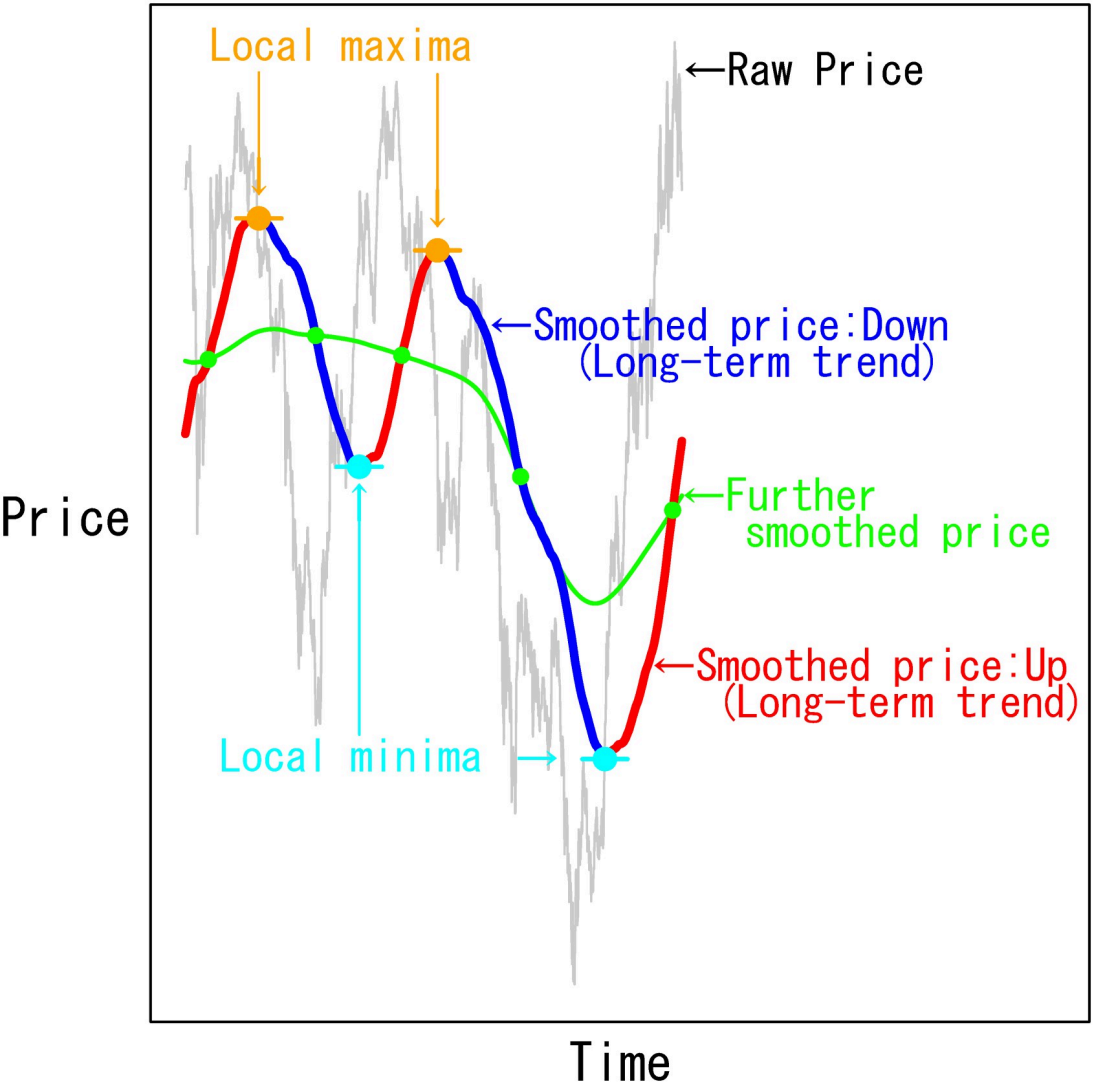
Algorithm to assign the smoothed price trend direction labels. Local maxima (minima) between adjacent intersections of the smoothed price trend (thick red and blue lines) and further smoothed price trend (green line) are set as the trend direction reversal points of the smoothed price.

Fig 2 illustrates the algorithm that assigns the trend direction class label. The gray line illustrates the raw price series, *P*. We calculate the *d*_*L*_-day simple MA-smoothed price up to time *t*, *y*_*t*_, as in Eq (2), and plot *y* = {*y*_1_, *y*_2_, ⋯, *y*_*t*_} as the thick red and blue lines. We obtain the green line, $\tilde{y}$, by further smoothing *y* to locate the local minima and maxima of *y*. We further smooth the line by applying the *d*_*f*_-day MA extended toward both sides via a Nadaraya-Watson kernel regression [43] with a box kernel of bandwidth *d*_*f*_. Along the smoothed trend line, *y*, the highest (lowest) point in orange (light blue) between the adjacent green intersections is the local maximum (minimum). Then, we assign a section from the local minimum (maximum) to the local maximum (minimum) as an “Up” (“Down”) direction class, as colored in red (blue).

The class assignment algorithm above is inspired by the MA-based technical indicators, the golden cross and the death cross, but we modify them such that the buy/sell signal timing is on the trend reversal point of the smoothed price to learn the market status by the trend direction class. We control the target scale of trend reversals by the averaging window, *d*_*L*_, which ranges from several days to several years.

#### 3.2.4 Input vectors to sensitively capture market condition changes

Given the smoothed price at time *t*, *y*_*t*_, as in Eq (2), we formulate the daily price change at time *t* as Δ*y*_*t*_ = log *y*_*t*_ − log *y*_*t*−1_. Let *x* at time step *t* be the smoothed price change series during *d*_*m*_ days such that $x(t,d_{m})=\{\Delta y_{t-d_{m}+1},\ldots ,\Delta y_{t}\}$. We use the recent moment statistics of *x*(*t*, *d*_*m*_) as input vectors, where the statistics are centralized about the mean for orders larger than one. The optimum order of the moments to use depends on the nature of the target movement and the available information. We focus on the orders up to four, the mean, variance, skewness, and kurtosis to avoid overfitting and to simplify the interpretation of the results.

The advantages of using the moment statistics of the target series as input vectors are two-fold. First, it requires no external data and model assumptions, avoiding the risk of model-misspecification due to market condition changes. Using the minimum number of input vectors is desirable to create the simplest model possible to mitigate the minority class data insufficiency problem. However, selecting the appropriate input vectors is a difficult task requiring expert knowledge of the target’s behavior. Moreover, the best set of input vectors can change over time, which requires frequent updates. In our approach, instead of renewing the optimum set of input vectors to adjust for the changes in market conditions, we capture the market status change itself directly by the changes in the moment statistic.

Second, moment statistics summarize certain characteristics of the probability density shape patterns and thus provide an effective warning of trend reversals by directly capturing the non-stationary market condition. In particular, the third moment, skewness, indicates the degree of trend direction class imbalance. Various streams of research provide evidence of the effective warning ability of moment statistics. The literature on anomalies in prices reports evidence that variance and skewness contain information on future return movements and offer predictability (see, e.g., [44]). Further, several research streams report the effectiveness of moment statistics as completely data-driven signals of the approach of critical transitions, such as ecology, neuroscience, and medicine. Several more detailed reviews are provided by [45–47].

#### 3.2.5 Long memory and remedies for class imbalance

Validation system is the *k*-fold blocked cross-validation of [48], because it is advantageous in time-series direction forecasting with small sample data compared to other procedures such as the standard out-of-sample procedure [49]. Parameter optimization is by a grid search.

All available data are partitioned into in-set samples used for training and out-of-sample used only to evaluate performance. In-set samples are partitioned into *k* sections of equal size. In the in-set training process, one block is withheld, as the in-set testing block, and the remaining *k* − 1 blocks are used for learning. Each of the *k* in-set blocks can be the in-set test block in the in-set training phase in turn. The directional accuracy of the candidate models is measured *k* times, and the average value of the *k* accuracy results is compared with candidate models to select the best model specification. The selected best model with the optimum parameter set is retrained using all *k* in-set training blocks to forecast the trend direction of the out-of-sample test block, which is used to evaluate trading performance.

In online learning environment, the training data period expands every time SVM is reconstructed, adding new data to use the full length, and partitioned into *k* sections of equal longer size. Adaptive block size is desirable to adjust to the drift length, and an appropriate fixed block size is a compromise between fast adaptivity (small size) and good generalization (large size) [30]. However, the size or variability of the respective block depends on the applications in which it is used [50]. For our research aim, loss aversion from large long-term trend reversals which rarely happens, it is critically important to hold long memory by holding longest available training data, which is also suggested by our preliminary test results.

The choice of model evaluation measure is especially important for avoiding trend reversal risks, particularly when the target data have concept drift with class imbalance. In stock markets, a high degree of class imbalance occurs in periods of stable upward trend phases. From the risk management perspective, risky events to avoid occur only rarely, but the correct detection of minor risky classes is more important than for non-risky classes. However, in the stock price trend direction classification problem, standard measures such as accuracy, evaluate the classification performance giving more weight to the relatively frequent, non-risky positive direction. Moreover, investors and traders tend to prioritize avoiding losses over seeking the same size gains [51]. Hence, we use an evaluation measure that places greater weight on the minority downward trend direction class for maximum exploitation of information about the downward trend class. Among these measures, we employ kappa statistics, which is one of the best evaluation measures receiving increased attention, particularly in the literature on online learning with concept drift and class imbalance [31, 32].

The specification of the directional evaluation measure, the kappa statistics, is as follows. Denote the true positive, false positive, true negative, and false negative as TP, FP, TN, and FN, respectively. Then, TP (FP) implies a correct (wrong) upward direction forecast; that is, a correct (wrong) buy decision. TN (FN) implies a correct (wrong) downward direction forecast; that is, a correct (wrong) sell decision. Accuracy is defined as

$$\begin{array}{c} Accuracy=p_{0}=\frac{TP+TN}{TP+TN+FP+FN}. \end{array}$$ (5)

Then, Cohen’s kappa statistic is

$$\begin{array}{c} Kappa=\frac{p_{o}-p_{e}}{1-p_{e}},\;\;p_{e}=\frac{\left(TP+FN)(TP+FP)+(FP+TN)(FN+TN\right)}{{\left(TP+TN+FP+FN\right)}^{2}}, \end{array}$$ (6)

where *p*_*o*_ is the relative observed agreement identical to accuracy, and *p*_*e*_ is the expected agreement obtained when the same number of examples are observed in each class.

### 3.3 Standard machine-classification-based non-trend-following rule

We add a standard machine-classification-based non-trend-following rule, OSVM, to our comparison to contrast the differences between trend-following rules and non-trend-following rules. OSVM learns the direction of non-smoothed raw daily price change series Δ*P* following common manner in machine learning studies. The design of OSVM is formulated as identical with that of LTDC except that it classifies direction of *raw* price change series Δ*P* instead of that of *smoothed* price change series Δ*y*. Corresponding to the replacement of target direction series from Δ*y* to Δ*P*, the input vectors are also replaced from the moment statistics of Δ*y* during past *d*_*m*_ days to those of Δ*P* during past *d*_*m*_ days.

## 4 Data

### 4.1 Data description

Selecting the dataset is important for an appropriate performance evaluation of a trend-following trading strategy, and [52] warn of the danger of data-snooping bias and recommend using only major stock market indices with sufficient length to enable the dataset to include long-term trend reversal information. This study uses the daily closing prices of four major daily stock market indices: the NYSE, NASDAQ, TOPIX, and FTSEALL. The selected indices are the weighted averages of all (or a major part of) the listed companies in each market. These are considered the most representative stock indices of the major countries. We collected these data from Datastream database for the period from 1973 to 2019. For all four stock indices, the in-sample trading period starts from January 1975, and the corresponding in-sample input data period starts from around the beginning of May 1973, which is the maximum averaging window 400 trading days before January 1975. The out-of-sample test period is from January 1995 to December 2019. This period covers the lower, unstable profitability of trend-following rules in the U.S. equity market, especially for large stock and market indices [13, 20–22].

**Fig 3:**
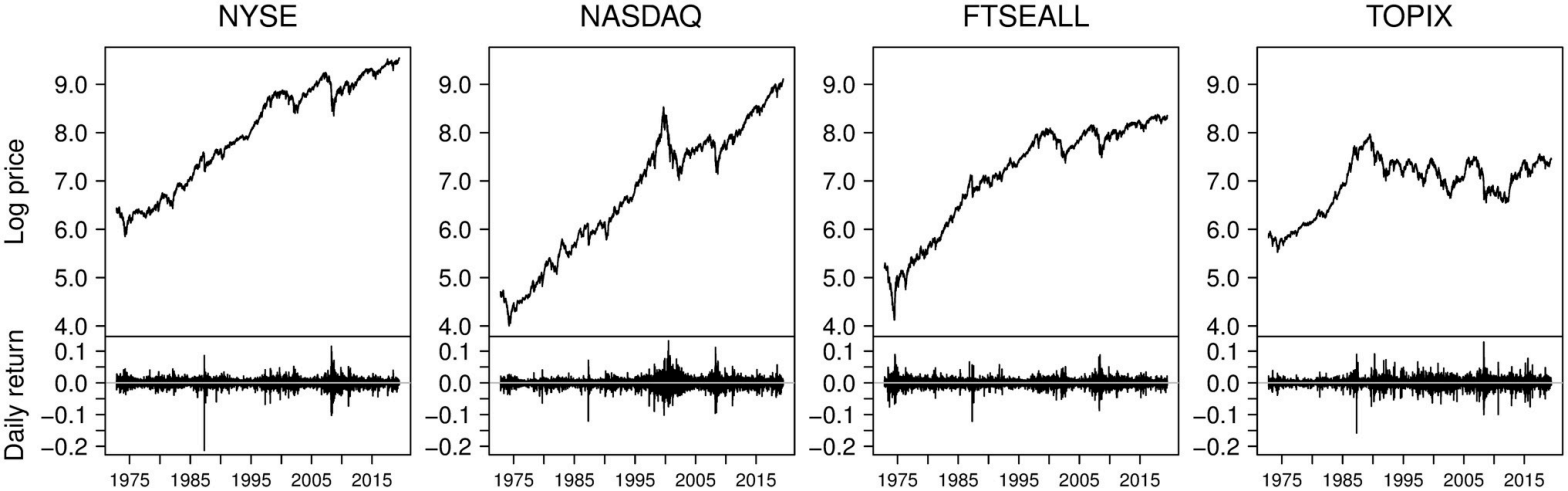
Daily log price and return time series of the NYSE, NASDAQ, FTSEALL, and TOPIX.

Fig 3 shows the movement of the log price and the corresponding daily returns for our full sample period. All four stock index price series have repeated upward and downward trend formations at the micro- and macro-scales. In the return sequences, we see periodic volatility clustering. Table 1 summarizes the basic statistics of the daily raw data and moment statistics, in addition to monthly directions. From Fig 3, we see that the long-term trend behavior of the TOPIX is quite different from that of the other indices. If we consider the long-term linear trend line, after the 1990s, only the TOPIX exhibits a sideways trend, while the other indices show a globally upward trend. The monthly direction ratio of the TOPIX in Table 1 also has the highest downward ratio among the four indices, but the difference from the other indices is not that large.

**Table 1:** Descriptive statistics.

Stock index	Daily returns					Monthly direction	
	Sample size	Mean	Variance	Skewness	Kurtosis	Up (%)	Down (%)
NYSE	11769	0.00026	0.00011	-1.055	27.364	334 (59.7)	225 (40.3)
NASDAQ	11769	0.00037	0.00015	-0.306	12.327	334 (59.7)	225 (40.3)
FTSEALL	11794	0.00026	0.00011	-0.272	11.505	340 (60.8)	219 (39.2)
TOPIX	11496	0.00014	0.00013	-0.378	13.414	314 (56.1)	246 (43.9)

Full sample period from 1973 to 2019.

### 4.2 Trend direction ratio imbalance problem

We must note the serious effect of changing the target trend scale in the difficulty of addressing the trend direction identification problem. The price trend becomes longer-term as the averaging window, *d*_*L*_, increases to smooth the trend further, resulting in a more serious trend direction ratio imbalance. A large positive trend direction ratio leads to deficient information about downward trend direction, rendering the trend direction identification problem difficult to address.

Using the full sample period data from 1973 to 2019, Fig 4 illustrates the increases in the positive trend direction ratio as the target trend scale to measure directional (i.e., return frequency) changes, from daily to two-yearly, for all stock market indices. For the U.S. stock market indices, the positive direction ratio increases to over 80% at the two-yearly frequency, while it is at a level slightly above 50% at the one-day change direction. In particular, it is difficult to predict the trend reversal of large *d*_*L*_ after a prolonged period of continued upward direction because information on the downward direction class in the past data, used to construct the model for trading decisions, is insufficient. Among the four indices, the degree of positive/negative direction class imbalance is the smallest for the TOPIX, and the difference with other indices increases on longer time scales.

**Fig 4:**
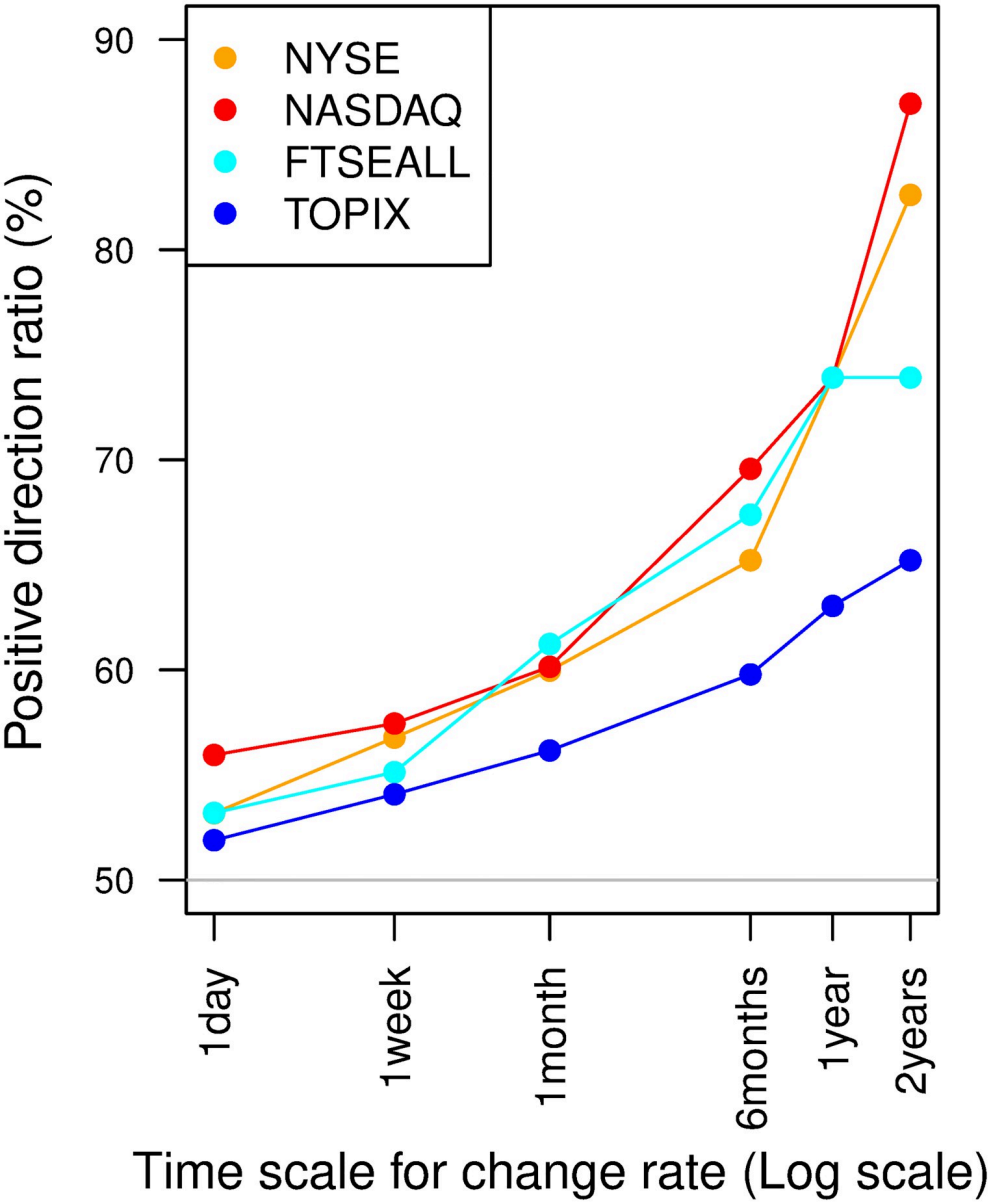
Changes in the positive direction ratio as the time scale of the change direction increases.

## 5 Research design

We next discuss the design and setting of the out-of-sample back test to compare four trading rules: TMOM, MACO, LTDC, and OSVM.

### 5.1 Computational set-ups

The TMOM and MACO rules are given in Eqs (1) and (3) with *z*_*t*_ = *P*_*t*_, respectively. We described the LTDC in Section 3.2 and OSVM in Section 3.3, respectively. We use the *ksmooth* function in R with bandwidth *d*_*f*_ to obtain the further smoothed price trend of LTDC, $\tilde{y}$, which we require for classifying the long-term trend direction. It is desirable that *d*_*f*_ changes as *d*_*L*_ changes, and we set *d*_*f*_ = 2*d*_*L*_.

As for the SVM classification of OSVM and LTDC, the computational details are as follows. We choose the short-term memory parameter for obtaining the moment statistics from *d*_*m*_ = {5, 10, 15, 20} days. We implement the SVM using the kernlab [53] package interface for libsvm [54], which includes an efficient version of the sequential minimization-optimization algorithm. For the nonlinear mapping *ϕ* from the input space to the high-dimensional feature space, we use the Gaussian radial basis function (RBF) kernel, which is the most suitable for our purpose and is widely applied to unknown general time series data. The soft margin penalty *C* controls the trade-off between the size of the margin and hyperplane violations. The RBF kernel requires parameter *γ*, which controls the influential range of a single training example. We set *C* = {2^−3^, …, 2^3^} and *γ* = {2^−3^, …, 2^3^}. The number of in-set blocks *k* is set as four.

### 5.2 Experimental scheme

The four trading rules make buy/sell trade decisions given the past daily price data. We evaluate the one-month ahead out-of-sample trading performance. We consider a monthly trading decision, as a one-month holding period achieved the best trading performance in a study by [11]. We evaluate the results using a prequential approach, which is desirable in a streaming setting with evolving data [7, 33]. Considering the loss aversion from long-term trend reversals, such as a stock market crash, we use an expanding (landmark) window that enables us to exploit the maximum information in the data.

The start of the in-sample trade performance evaluation period is 1975.1, and the input data period starts from 400 trading days prior to obtain Eqs (1) and (2) with the maximum averaging window. The sliding out-of-sample test period starts from 1995.1 and ends in 2019.12, during which previous studies report an insignificant profitability of trend-following rules. A buy/sell decision for the trade at 1995.1 is made after 1994.12 based on the models using in-sample training data up to 1994.12. We evaluate the out-of-sample one-month returns from 1994.12 to 1995.1. The buy/sell decision of the second test month, from 1995.1 to 1995.2, is made based on the in-sample data up to 1995.1, and the same applies to the following months.

For the SVM classification of OSVM and LTDC, the model is reconstructed every five years, which is determined as a compromise between computational cost and model durability under the constraint of available data. The construction of the first model is based on the data up to 1994.12 and the first (second) monthly trade decision for 1995.1 (1995.2) is made when the input vector up to 1994.12 (1995.1) becomes available. The application of the first model continues until the monthly trade decision for 1999.12. The second model is constructed using data up to 1999.12, and the first (second) monthly decision for trade from 1999.12 (2000.1) to 2000.1 (2000.2) occurs in 1999.12 (2000.1). This process continues until the last test month, 2019.12, where we apply the fifth model using data up to 2014.12 and the input vector up to 2019.11 for the prediction.

The subsequent section compares the out-of-sample test results of the four trading rules from 1995.1 to 2019.12 to analyze the causes of the unstable profitability of trend-following rules and explore the potential remedies.

## 6 Results

### 6.1 Unconditional performance analysis

#### 6.1.1 Profitability changes by target index and averaging window

**Fig 5:**
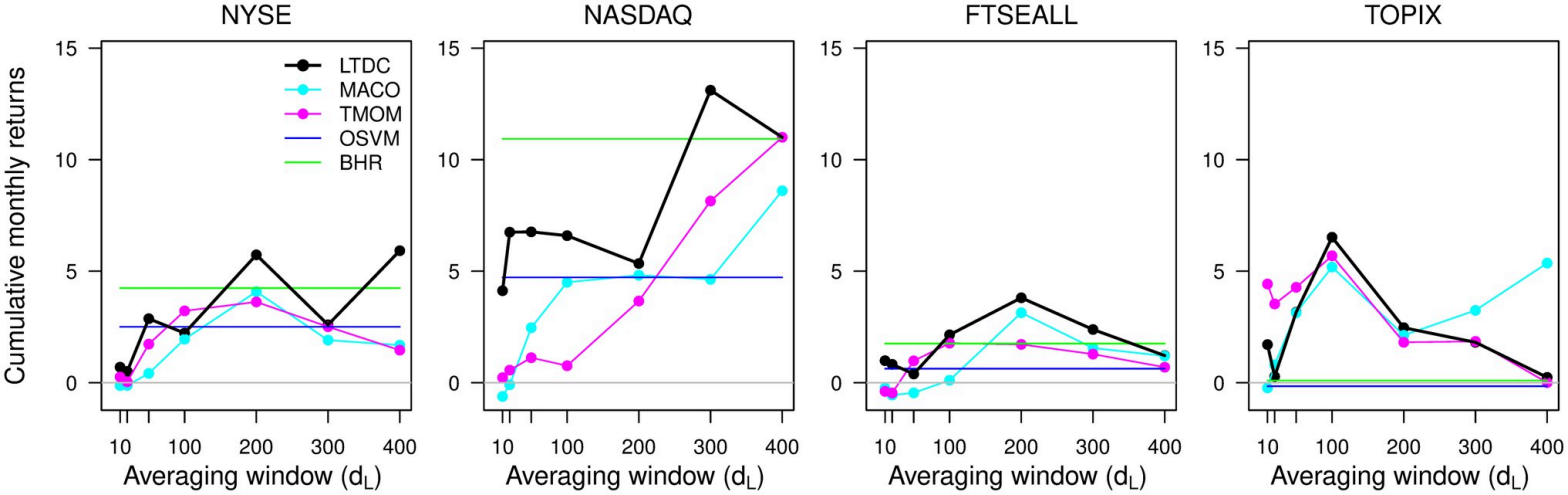
Cumulative monthly returns by market and averaging window.

The out-of-sample comparative study shows that changing the target index and averaging window, *d*_*L*_, has a significant effect on unconditional trading performance, and we see common patterns for the trend-following rules. Fig 5 shows the cumulative monthly returns of the trend-following strategies by trading four major stock market indices in the recent period with different *d*_*L*_ lengths. We find that three trend-following rules, MACO, TMOM, and LTDC, mostly outperform BHR for the TOPIX, but their performance for the other indices depends on *d*_*L*_. Moreover, the excess return of the BHR of trend-following rules is higher for lower BHR indices, and the optimum *d*_*L*_ for each index is mostly common among the trend-following rules. Thus, the effect of changing the trading rule is sometimes outweighed by the change in the target index or *d*_*L*_. Among the four rules, the performance of LTDC is best mostly around the common optimum *d*_*L*_, with no negative returns for all indices and *d*_*L*_.

**Fig 6:**
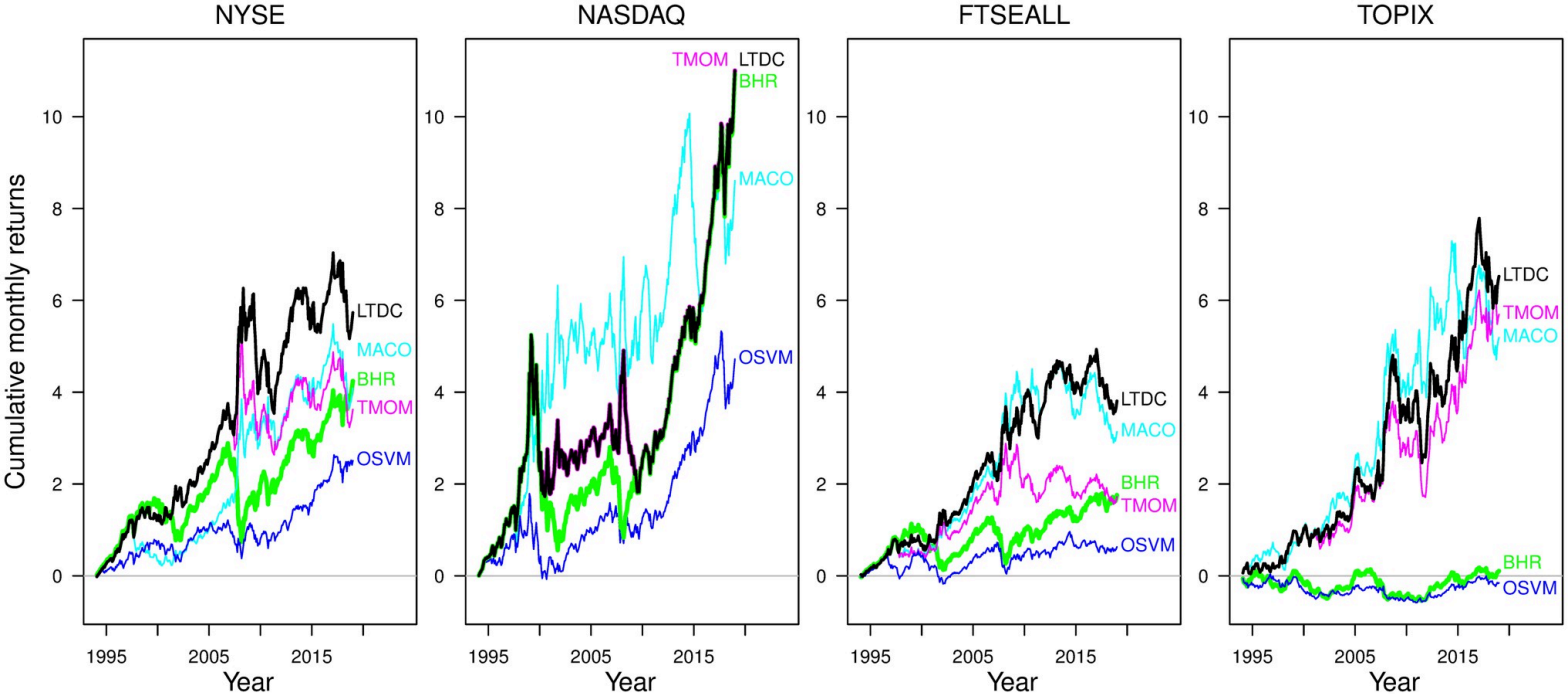
Evolution of cumulative monthly returns at the common optimum averaging window for all trend-following rules. The selected common optimum averaging window is *d*_*L*_ = 400 for NASDAQ, 200 for the NYSE and FTSEALL, and 100 for the TOPIX.

Selecting the averaging window appropriate for each market property is quite effective for trend-following rules to significantly outperform BHR by avoiding losses from trend reversals. Fig 6 illustrates the evolution of the cumulative returns of trend-following strategies with respect to the common optimum *d*_*L*_, wherein the average of all three trend-following rules’ returns are maximized: *d*_*L*_ = 400 for NASDAQ, 200 for NYSE and FTSEALL, and 100 for TOPIX. We find that the machine-classification-based non-trend-following rule, OSVM, performs poorly in most cases, significantly underperforming the trend-following rules, especially for the TOPIX. The performance differences between the trend-following rules and BHR widens notably during a large price decline, implying the common advantage of trend-following rules in avoiding losses from trend reversals. As a result of the significant effect of market trend conditions common to trend-following rules, we observe the coincidence of the trading decision of LTDC and TMOM for the NASDAQ with *d*_*L*_ = 400. In our experiment, this coincidence occurs only with the combination of the NASDAQ with *d*_*L*_ = 400, which has the simplest classification problem. We presume that the lowest noise level of the 400-day MA direction makes the LTDC learning (by matching return moments) degenerate to focus only on the past price mean movement. The LTDC has the best performance of all methods.

The features illustrated in Figs 5 and 6 are in line with results reported by [13] and [29] in that the TMOM and MA-based rules have many similarities in their trading behavior. However, their finding that the MA-based rules outperform the TMOM rule is not clearly supported: we observe that the selection of *d*_*L*_ and the market conditions affect the relative performance of the TMOM and MACO. Alternatively, we find significantly different behavior between the three trend-following rules and the non-trend-following rule. This result suggests that the similarity and the nature common to trend-following rules stem from the focus on the direction of the *long-term price trend change* instead of the *monthly raw price change*, as it is the only difference between the OSVM and LTDC.

#### 6.1.2 Downside risk protection

Our results validate the significant ability to protect against downside risks rather than high volatility as a property common to trend-following rules. We evaluate the risk tolerance of each rule by the standard risk measures: Sharpe ratio, Sortino ratio, and maximum drawdown. We set the risk-free rate at zero. Table 2 summarizes the risk tolerance statistics of the out-of-sample test period with reality-check significance by [55], corresponding to the hypothesis that the performance measures outperform those of BHR, where we use the stationary block bootstrap method of [56] with the optimum block length determination by [57]. High volatility significantly affects the unstable performance of trend-following rules [13, 17, 18]. However, Table 2 shows that the performance of trend-following rules relative to the BHR is best for maximum drawdown, and their Sortino ratios are better than their Sharpe ratios. Most trend-following results for the lowest BHR market, TOPIX, are significant, indicating that trend-following rules are advantageous for such stagnating (i.e., not stably upward) market conditions. In addition, the *d*_*L*_ minimizing maximum drawdown is the same or smaller than the return-maximizing *d*_*L*_. For all risk indicators, the LTDC has the best overall performance, especially at around the optimum *d*_*L*_, where it outperforms the BHR, suggesting the effectiveness of incorporating machine classification-based solutions to avoid downside risks. OSVM offers significant drawdown risk protection for the NYSE and NASDAQ, whose cause we discuss in more detail later.

**Table 2:** Risk tolerance statistics.

Stock index	*d*_*L*_	Sharpe Ratio^(1)^					Sortino Ratio^(2)^					Maximum drawdown^(3)^				
		LTDC	MACO	TMOM	OSVM	BHR	LTDC	MACO	TMOM	OSVM	BHR	LTDC	MACO	TMOM	OSVM	BHR
NYSE	10	0.219	0.035	0.135	0.420	0.534	0.312	0.050	0.204	0.621	0.767	0.667	0.549	0.637	0.376**	0.552
	20	0.187	0.037	0.084			0.260	0.053	0.123			0.483***	0.633	0.524		
	50	0.447	0.168	0.349			0.672	0.249	0.540			0.465	0.497	0.323***		
	100	0.396	0.371	0.471			0.597	0.574	0.723			0.578	0.316***	0.472		
	200	0.604	0.524	0.497			0.926	0.795	0.745			0.375**	0.449	0.441*		
	300	0.427	0.367	0.420			0.648	0.538	0.626			0.429*	0.569	0.514		
	400	0.611	0.345	0.321			1.001	0.506	0.476			0.293***	0.466*	0.585		
NASDAQ	10	0.405	−0.056	0.150	0.426	0.560	0.606	−0.079	0.216	0.620	0.836	0.757	0.853	0.768	0.667**	0.750
	20	0.481	0.097	0.192			0.711	0.140	0.287			0.750	0.705*	0.602**		
	50	0.479	0.334	0.246			0.739	0.502	0.369			0.493***	0.502***	0.559***		
	100	0.476	0.417	0.213			0.719	0.649	0.311			0.460***	0.441***	0.706		
	200	0.443	0.427	0.386			0.674	0.656	0.598			0.459***	0.558***	0.458***		
	300	0.589	0.420	0.510			0.937	0.657	0.791			0.538***	0.550***	0.543***		
	400	0.560	0.518	0.560			0.859	0.822	0.859			0.564***	0.393***	0.564***		
FTSEALL	10	0.276	−0.017	−0.083	0.215	0.377	0.400	−0.024	−0.116	0.294	0.527	0.345**	0.504	0.618	0.463	0.469
	20	0.250	−0.179	−0.120			0.346	−0.248	−0.166			0.462	0.634	0.656		
	50	0.167	−0.118	0.274			0.245	−0.164	0.425			0.450	0.617	0.337*		
	100	0.417	0.098	0.377			0.635	0.146	0.593			0.333*	0.503	0.341*		
	200	0.549	0.502	0.371			0.867	0.801	0.565			0.237***	0.311*	0.340*		
	300	0.439	0.352	0.317			0.690	0.534	0.487			0.319*	0.443	0.495		
	400	0.308	0.306	0.226			0.470	0.462	0.338			0.395	0.466	0.571		
TOPIX	10	0.317	0.027	0.478*	0.050	0.111	0.465	0.038	0.757*	0.069	0.155	0.492	0.599	0.334**	0.599	0.595
	20	0.143	0.223	0.435			0.212	0.333	0.695			0.475	0.421*	0.394		
	50	0.416	0.415	0.471*			0.648	0.641	0.741*			0.336**	0.352*	0.433		
	100	0.555*	0.509*	0.527*			0.885*	0.797*	0.840*			0.404	0.348*	0.434		
	200	0.373	0.350	0.325			0.585	0.528	0.501			0.492	0.629	0.554		
	300	0.325	0.420	0.328			0.498	0.659	0.503			0.460	0.359*	0.460		
	400	0.136	0.516	0.089			0.199	0.820	0.128			0.565	0.387	0.584		

(1) and (2) are annualized. (3) is the maximum drawdown from the peak. The reality check corresponds to the hypothesis that the performance measures outperform those of the BHR. Statistical significance at the 1%, 5%, and 10% levels are indicated by ***, **, and *, respectively.

**Fig 7:**
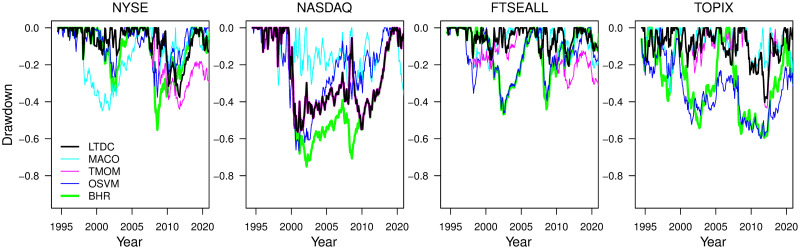
Time series plot of the drawdown at the *d*_*L*_ common optimum for all trend-following rules.

**Fig 8:**
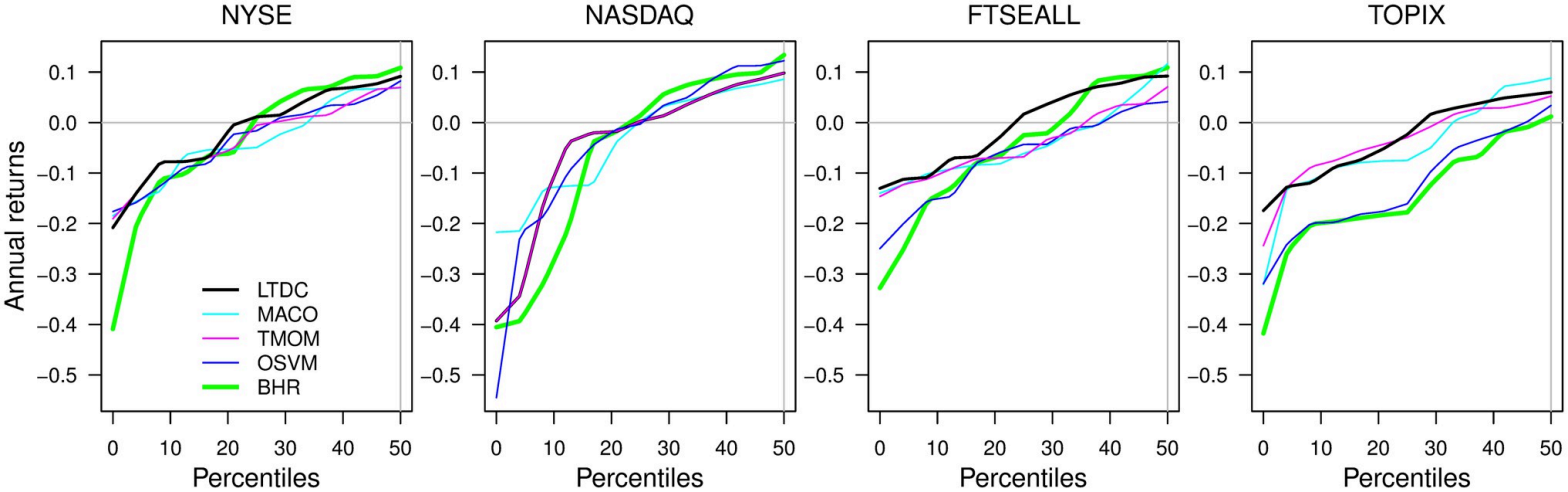
Quantile points of negative returns at the *d*_*L*_ common optimum for all trend-following rules.

The stability of downside risk aversion of the LTDC is the best among the four rules from Figs 7 and 8, implying the effectiveness of trend-following with remedies for class imbalance and non-stationarity to avoid downside risks. Fig 7 depicts the time series plot of the drawdown for each rule since the start of the test period in 1995.1 at the *d*_*L*_ that maximizes the average returns of the three trend-following rules, as in Fig 6. Compared to the BHR (green line), the drawdown size of the LTDC (bold black line) is significantly smaller most of the time, exhibiting a stable drawdown protection ability. This is in contrast to the other rules, which experience periods with significantly underperforming BHR for some indices. Fig 8 compares the quantiles of the annual negative returns at the *d*_*L*_ that maximizes the average returns of the three trend-following rules. In Fig 8, the trend-following rules are mostly above the BHR, while the OSVM (blue line) is mostly overlapping the BHR, except for the NYSE and NASDAQ, whose positive direction ratio is the first and second among the four indices, respectively. Most of the time, the LTDC has the smallest loss, especially in terms of the largest downside risk.

#### 6.1.3 Performance robustness

LTDC is robust in the mis-selection of the averaging window size and realizes an enhanced adaptation to market condition changes. Authors in [29] emphasize the difficulty of selecting the time-varying return-maximizing averaging window, and show that the forecast accuracy of trend-following rules depends on the robustness to changes in *d*_*L*_. They propose a measure of performance robustness based on the average Sharpe ratio over all averaging window sizes. We follow their approach to obtain an indicator for robustness to the change in the averaging window size, expanding the set of target risk measures.

Table 3 reports the Sharpe ratio, Sortino ratio, and maximum drawdown averaged over all *d*_*L*_ values of *d*_*L*_ = 10, 20, 50, 100, 200, 300, and 400. We test the equality of the *d*_*L*_ average of the risk statistics for each pair of rules. Table 3 also presents the reality check p-values following [55] based on the stationary block bootstrap by [56, 57]. Table 3 shows that the LTDC method has the best overall robustness to the change in *d*_*L*_, with only minor performance fluctuations among the target indices. With respect to the Sharpe and Sortino ratios, the LTDC outperforms the rules for all indices except the TOPIX. Regarding maximum drawdown, the performance robustness of the trend-following rules is mostly stable for all target indices, in contrast to the OSVM, which differs considerably for each index.

**Table 3:** Average risk tolerance statistics and the tests of equality comparison between two rules.

Stock index		Trading rule			
		LTDC	MACO	TMOM	OSVM
*Average Sharpe ratio over all d_L_*					
NYSE	Average Sharpe ratio P-value	0.413	0.264	0.325	0.420
		LTDC	0.232	0.410	0.978
		MACO	NA	0.320	0.498
		TMOM	NA	NA	0.668
NASDAQ	Average Sharpe ratio P-value	0.491	0.308	0.322	0.426
		LTDC	0.154	0.102	0.664
		MACO	NA	0.840	0.610
		TMOM	NA	NA	0.636
FTSEALL	Average Sharpe ratio P-value	0.344	0.135	0.195	0.215
		LTDC	0.072*	0.124	0.592
		MACO	NA	0.278	0.756
		TMOM	NA	NA	0.916
TOPIX	Average Sharpe ratio P-value	0.324	0.351	0.379	0.050
		LTDC	0.700	0.312	0.220
		MACO	NA	0.628	0.202
		TMOM	NA	NA	0.170
*Average Sortino ratio over all d*_*L*_					
NYSE	Average Sortino ratio P-value	0.631	0.395	0.491	0.621
		LTDC	0.224	0.408	0.996
		MACO	NA	0.358	0.540
		TMOM	NA	NA	0.706
NASDAQ	Average Sortino ratio P-value	0.749	0.478	0.490	0.620
		LTDC	0.228	0.160	0.610
		MACO	NA	0.932	0.712
		TMOM	NA	NA	0.688
FTSEALL	Average Sortino ratio P-value	0.522	0.215	0.304	0.294
		LTDC	0.086*	0.158	0.546
		MACO	NA	0.296	0.832
		TMOM	NA	NA	0.976
TOPIX	Average Sortino ratio P-value	0.499	0.545	0.595	0.069
		LTDC	0.680	0.302	0.216
		MACO	NA	0.612	0.200
		TMOM	NA	NA	0.176
*Average maximum drawdowns over all d*_*L*_					
NYSE	Average maximum drawdowns P-value	0.470	0.497	0.499	0.376
		LTDC	0.978	0.676	0.114
		MACO	NA	0.522	0.228
		TMOM	NA	NA	0.122
NASDAQ	Average maximum drawdowns P-value	0.574	0.572	0.600	0.667
		LTDC	0.350	0.906	0.144
		MACO	NA	0.146	0.116
		TMOM	NA	NA	0.232
FTSEALL	Average maximum drawdowns P-value	0.363	0.497	0.480	0.463
		LTDC	0.020**	0.006***	0.350
		MACO	NA	0.968	0.784
		TMOM	NA	NA	0.822
TOPIX	Average maximum drawdowns P-value	0.460	0.442	0.456	0.599
		LTDC	0.796	0.612	0.340
		MACO	NA	0.504	0.294
		TMOM	NA	NA	0.472

The hypotheses that the Sharpe and Sortino ratios (maximum drawdown) are higher (lower) than those of the BHR is tested using a reality check.

***, **, and * indicate statistically significant reality check p-values at the 1%, 5%, and 10% levels, respectively.

The LTDC has a significant advantage in terms of robustness for the FTSEALL. Trend-following rules are commonly advantageous for the TOPIX. On the other hand, the OSVM tends to be advantageous for the NYSE and NASDAQ, except for the maximum drawdown of NASDAQ. Fig 4 shows the positive trend direction ratios for the NYSE, NASDAQ, FTSEALL, and TOPIX, in that order. Hence, the relative performance patterns are consistent with our conjecture that high positive trend direction ratios exacerbate the performance of trend-following rules, which we subsequently validate. The validity of this conjecture implies that the outperformance of the LTDC over the MACO and TMOM for FTSEALL suggests a significant improvement in dealing with the class imbalance problem by combining machine classification with trend-following rules to improve the adaptation of the rule to varying market conditions.

### 6.2 The mechanism of performance instability

We focus on the trend direction phase as a significant factor in the unstable profitability of trend-following rules. Among the potential market influences, [29] theoretically demonstrate the significant effect of trend strength and the averaging window, assuming an autoregressive return process. Moreover, they emphasize the importance of empirically estimating the trend strength for improving the trading performance under uncertainty. We consider the BHR during a calendar year as a simple measure of trend direction and strength, computable solely from price series.

#### 6.2.1 Effect of trend direction phase on trade decision accuracy

**Fig 9:**
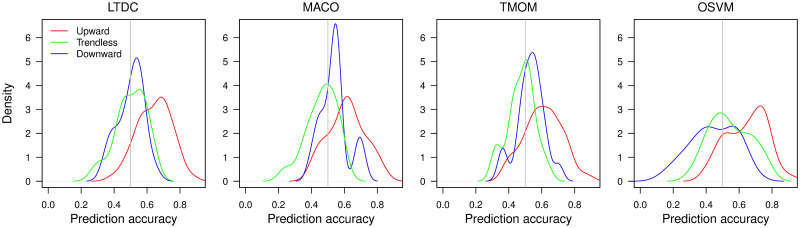
Kernel density estimates of price direction prediction accuracy by trend direction phase. Trend direction is classified according to yearly BHRs with the threshold at ±0.1. The accuracy of trend-following rules is averaged over all *d*_*L*_ values.

The difficulty of the buy/sell decision changes according to the trend *direction* phase, and the most difficult phase differs for trend-following and non-trend-following rules. Fig 9 shows the kernel density estimates of the trade decision accuracy of each trading rule classified by the three trend direction phases of the target price index: positive, negative, and trendless. Using 25 years of trading decisions in the out-of-sample test results, the years with higher (lower) annual market returns (BHR) than 0.1(−0.1) are classified as a positive (negative) trend phase, and those within ±0.1 are classified as a trendless phase. We compute the accuracy of the trading decisions in a calendar year using Eq (5) for each *d*_*L*_ and index, evaluating the correctness of 12 monthly trading decisions given the target actual raw monthly price change directions. In a strict sense, evaluating the accuracy of predicting the direction of *raw* price changes is not a fair comparison, since the target direction to predict is that of the *raw* price change for the non-trend-following rule, but for the three trend-following rules, it is that of the *smoothed* trend. We take the average accuracy of the trend-following rules across all *d*_*L*_ values.

In Fig 9, the prediction accuracy in the positive trend direction phase (red) is located at the extreme right for all trading rules, implying the highest accuracy phase, or the easiest phase for which to predict the direction. However, the most difficult phase to predict the monthly trend direction differs for the trend-following rules and non-trend-following OSVM. The phase located at the extreme left is the trendless phase (green) for the trend-following rules, TMOM, MACO, and LTDC, but it is the downward trend phase (blue) for the non-trend-following OSVM. The relative difficulty of the trendless phase and downward phase for the LTDC is lesser than that of the other trend-following rules, possibly because it combines the properties of OSVM and trend-following rules.

#### 6.2.2 Discrepancies in the optimum averaging window between risk phases

**Fig 10:**
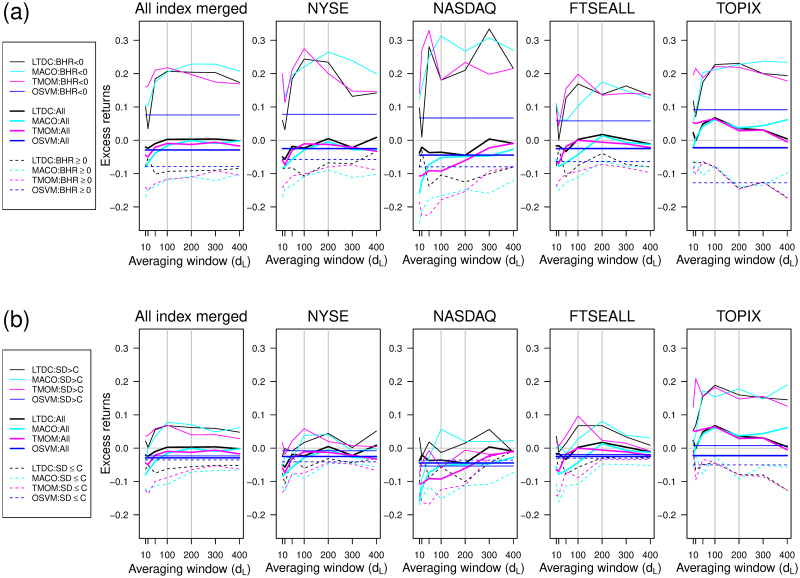
Changes in the relationship between annual excess returns and averaging window by market risk condition. (a)Risk phase by market trend direction; (b) Risk phase by market volatility level.

While market stress, such as downside risks and high volatility, influence the performance of trend-following rules [13, 15, 29, 58], how those factors affect unstable profitability is not yet clear. Fig 10 comprehensively illustrates the effect of such market risk conditions on the relationship between excess returns relative to the BHR and averaging window. We measure the market risk condition by the market trend direction sign or the market volatility level.

In Fig 10(a), we split the annual excess returns of each rule by the trend direction of the target stock price index in a calendar year; that is, the sign of the yearly BHR. Similarly, we divide the annual excess returns of each rule into two phases at *s*.*d*. = *C* to plot Fig 10(b), wherein *C* is the median of the standard deviation of each stock index. The horizontal axis of Fig 10 is the averaging window size, *d*_*L*_, which we interpret as the trend scale of the direction to predict. The bold solid lines indicate the unconditional excess returns, while the thin solid and dotted lines indicate the risky and non-risky phases, respectively. Black, light blue, pink, and blue denote the LTDC, MACO, TMOM, and OSVM, respectively. The leftmost panels of Fig 10(a) and 10(b) merge the results for the four indices on their respective right side, summarizing the effect of changing *d*_*L*_ on the profitability of trend-following rules by trend direction phase.

From Fig 10(a), we find discrepancies in the optimal averaging window of the trend-following rules between the positive and negative trend direction phases. As the leftmost merged panel shows, the averaging window affects the excess return differently for each trend direction phase, and the significant outperformance in the negative trend direction phase compensates for the under-performance in the positive trend direction phase. This result is in line with the reported advantages of trend-following rules during market stress periods. The relationship between *d*_*L*_ and excess returns in the negative trend phase is an inverted U-shaped curve, whereas that in the positive trend phase is rising to the right. Hence, the optimal *d*_*L*_ tends to be smaller than that for the positive trend direction phase. According to [10, 11], setting a longer investment horizon, a larger *d*_*L*_ in our context, is more profitable, but our result suggests that it is only valid during a period matching a positive trend direction phase.

The performance of each trading rule varies for each stock market index. This varying pattern could be explained by the degree of discrepancy by trend direction phase, where the optimal *d*_*L*_ in the positive trend direction phase is critical. The relationship between *d*_*L*_ and the excess returns of trend-following rules in the positive market return (BHR) phase (dotted lines) is upward trending for the NYSE and NASDAQ, gently inverted V-shaped for the FTSEALL, and downward-trending for the TOPIX. The difference between the optimal *d*_*L*_ in each trend direction phase is the largest for the NYSE and NASDAQ, followed by the FTSEALL and the TOPIX. Hence, it becomes more difficult to stay near the optimal *d*_*L*_ at trend reversals for the NYSE and NASDAQ than for the TOPIX. That is, dealing with sudden trend reversals, which is the problem of non-stationarity, is difficult for trend-following rules in trading the NYSE and NASDAQ relative to trading the TOPIX.

We argue that the large discrepancy of the optimum *d*_*L*_ size by trend direction phase is due to the stable long upward trend period, which we can empirically measure by the positive trend direction ratio. With respect to the out-of-sample test period from 1995 to 2019, the average positive/negative return ratios of the yearly trend direction of the NYSE, NASDAQ, FTSEALL, and TOPIX are 76%, 76%, 68%, and 52%, respectively.

For the target index series with a largely positive trend direction, such as the NYSE and NASDAQ, trading decisions based on the past price movement are overwhelmed by the positive trend direction by frequency, which leads to a greater emphasis on the upward trend direction phase. This corresponds to the case when a long-term upward trend phase continues, and simply riding the upward trend without considering protection against downside risk can generate profit. Hence, a larger *d*_*L*_ becomes more profitable because it captures the long-term trend with less noise. However, adapting to trend reversals after a long continued positive trend is difficult, as traders are unprepared to detect the signs of a trend reversal. The situation is different for a balanced index such as the TOPIX, where the trend direction class imbalance problem is not severe. The downside risk of the TOPIX is larger than that of the other indices, even in a positive trend direction year, and maintaining protection against the downside risk, as in the negative trend direction phase at around *d*_*L*_ = 100, is desirable. With less discrepancy in the optimal *d*_*L*_, therefore, it is easier to maintain the optimal *d*_*L*_ for the TOPIX.

The relative advantage of the OSVM for the NYSE might be explained by the smaller imbalance in the target trend direction for trading decisions. The time scale of the target direction of the OSVM is smaller than that of trend-following rules. Fig 4 shows that the positive direction ratio of the NYSE and NASDAQ is more balanced for the shorter target time scale, which is one month for the OSVM but more than six months for the three trend-following rules. For the NYSE and NASDAQ, we conjecture that the benefit of reducing noise by targeting the smoothed trend direction is outweighed by the negative effects of the large class imbalance.

Table 4 reports the Pearson correlation and Spearman rank correlation test results for the risk factors and excess returns in the same year. These results confirm the adverse effect of high positive direction ratios on the excess returns of the timing strategies. We take the average excess returns of each trading rule in a year across all *d*_*L*_ values. For the first risk factor, the positive direction ratio, we use the monthly direction ratio in a year to ensure a sufficient sample size. We find a high significance of the positive direction ratio for all trading rules, while the OSVM has a lower significance with a smaller absolute coefficient than that for the trend-following rules. Given the statistically significant negative effect of the larger positive trend direction ratios on the performance of trend-following rules, the outperformance of the LTDC relative to the MACO and TMOM for the FTSEALL in the downside protection robustness result of Table 3 can be understood as the increased adaptability of trend-following rules to a higher degree of class imbalance, implying the effectiveness of machine classification-based remedies for class imbalance.

**Table 4:** Pearson and Spearman rank correlations between risk factors and excess returns averaged over all *d*_*L*_ values.

Risk factor	Method	Pearson correlation		Spearman rank correlation	
		Coefficient	P-value	Coefficient	P-value
Positive direction ratio^(1)^	LTDC	-0.655***	1.46e-13	-0.623***	4.55e-12
	MACO	-0.637***	1.06e-12	-0.568***	6.84e-10
	TMOM	-0.653***	1.74e-13	-0.626***	3.27e-12
	OSVM	-0.440***	4.56e-06	-0.494***	1.80e-07
Market volatility^(2)^	LTDC	0.561***	1.29e-09	0.256**	0.010
	MACO	0.563***	1.07e-09	0.224**	0.025
	TMOM	0.553***	2.39e-09	0.301***	0.002
	OSVM	0.198**	0.048	0.147	0.144

^(1)^ Positive monthly direction ratio of the stock index in a calendar year.

^(2)^ Standard deviation of daily index returns in a calendar year. Results for each index are merged.

***, **, and * indicate statistically significant p-values at the 1%, 5%, and 10% levels, respectively.

The findings from Fig 10(b) according to market volatility level are mostly like those of Fig 10(a) by market trend direction. However, the overall differences in performance by market, especially in a risky phase (upper side of the panels) are less obvious in Fig 10(b) than in Fig 10(a). Table 4 confirms the correlation between the volatility level of the index and the excess return of each rule, where the average daily standard deviation of each stock index in a year represents market volatility. However, the coefficients and significance are smaller than those of the positive direction ratio. This is consistent with the results in Tables 2 and 3, which indicate that the trend-following rules are advantageous in terms of downside risk rather than volatility risk. A large trend reversal tends to accompany a high volatility phase. While high volatility is a possible factor accounting for the unstable performance of trend-following rules, we find that trend direction and strength are likely to be more essential factors in the trading behavior of trend-following rules.

### 6.3 Towards optimization of the averaging window size

We see that trend direction change (non-stationarity) and the trend direction ratio (class imbalance) significantly affect the trading performance of trend-following rules. The profit-maximizing *d*_*L*_ should be smaller in a downward trend phase than in an upward trend phase to ensure the sensitivity to capture trend reversal signals. Given the difficulty of predicting sudden trend reversals, the key is to keep the averaging window near the optimal level that balances the trade-off between the trend and reversal to adapt to each trend direction phase.

**Fig 11:**
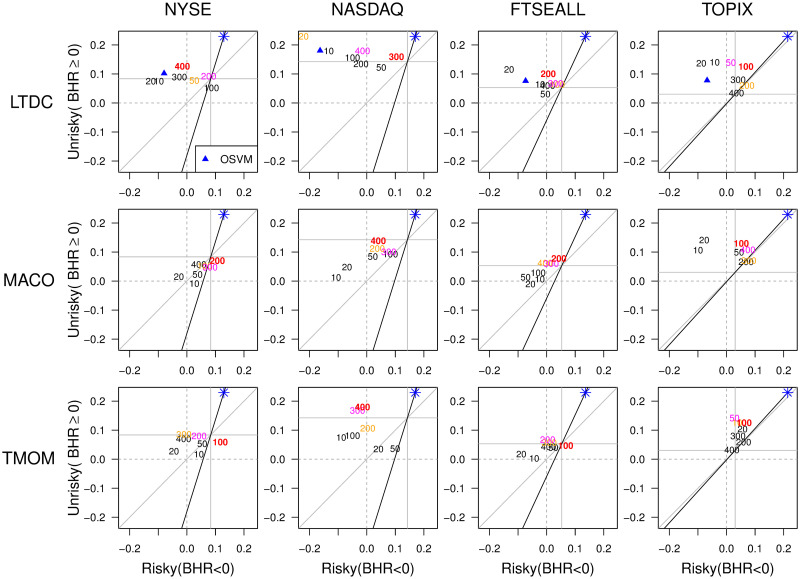
Averaging windows and the balance between positive and negative market directions. The red, pink, and orange figures are the first, second, and third profit-maximizing *d*_*L*_ values, respectively. The blue star at the upper right end of the black upward-sloping line indicates the direction for maximizing profit while keeping the upward/downward ratio of the corresponding market.

Fig 11 shows that the *d*_*L*_ that achieves the maximum trading profit best balances the trade-offs. The figure plots the 25-year out-of-sample results, where the horizontal (vertical) axis is the average annual returns when the yearly trend direction of the target index is negative (positive). The black upward-sloping line shows the 25-year average positive/negative ratio of yearly change direction of the target index, indicating the degree of the yearly trend direction class imbalance of the target index. The gray 45-degree lines indicate the even yearly trend direction ratio. The red, pink, and orange figures in each panel indicate the first, second, and third profit-maximizing *d*_*L*_ values, respectively. The blue star located at the upper right end of the black upward-sloping line indicates the direction that maximizes the profit while keeping the balance of the yearly trend direction ratio. It also indicates the overall strongest trend point along the trend direction balancing line, as the positive direction ratio tends to be larger than the negative direction ratio in stock markets. In Fig 11, the colored *d*_*L*_ points tend to be located closest to the blue stars, mostly in order by profit: red is the closest, followed by pink and orange. This result suggests that *trend strength* and the *trend direction ratio* are the key factors to consider when selecting the return-maximizing *d*_*L*_ best adapted to occasional trend direction phase changes; that is, non-stationarity.

**Fig 12:**
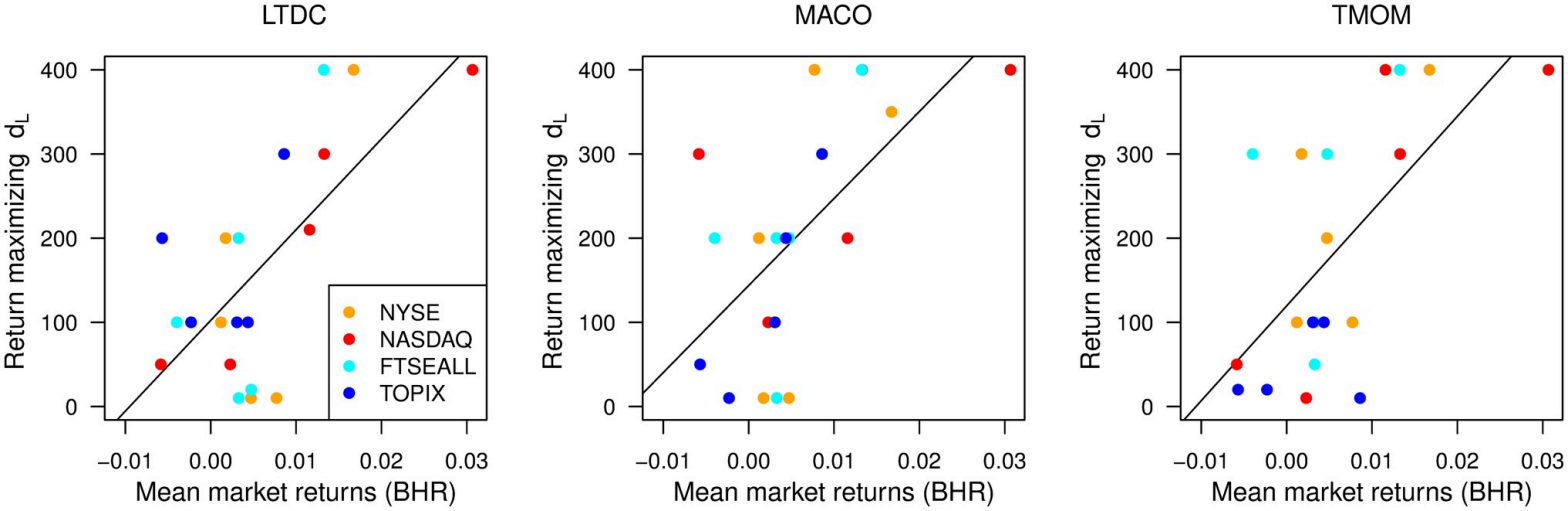
The profit-maximizing *d*_*L*_ value and mean market returns. In two-way ties, the mean *d*_*L*_ values are used.

We suggest the mean returns of the target price series as a simple guideline to select the optimal *d*_*L*_. The mean returns reflect the key information of both trend strength and the trend direction ratio. A stronger trend yields a larger mean return. The correlation between the monthly positive price direction ratio and mean returns in the same year is around 80% for each market. Fig 12 illustrates the relationship between the mean returns and the return-maximizing *d*_*L*_ value. We divide the 25 out-of-sample test years into five blocks (1995-1999, 2000-2004, 2005-2009, 2010-2014, and 2015-2019) and plot the mean returns during each block and the corresponding profit-maximizing *d*_*L*_ value. The orange, red, light blue, and blue points indicate the NYSE, NASDAQ, FTSEALL, and TOPIX, respectively. We find a positive relationship between mean returns and the profit-maximizing *d*_*L*_ value. The estimated coefficients of the simple regression lines in each panel are all significant, with p-values less than 1%.

Using this relation, we can compute a roughly appropriate *d*_*L*_ depending on the market condition, namely trend strength and the trend direction ratio. In the case of monthly trading by LTDC, for example, an appropriate *d*_*L*_ to use is determined by ${\hat{d_{L}}}^{*}=102.27+10781.68\;{\bar{r}}_{5y}$, where ${\bar{r}}_{5y}$ is the mean returns during the past five years. By incorporating this *d*_*L*_ determination step before SVM re-construction, we can implement LTDC trading with adaptive *d*_*L*_ conditional on market trend condition. Note that *d*_*L*_ must be determined *before* SVM re-construction, because *d*_*L*_ controls the target trend scale to classify. By executing the backtest, this method can be implemented to any trend-following rule to trade any price series. The realized past returns are a helpful first step in selecting the appropriate *d*_*L*_ value to maximize the trading performance of trend-following rules by keeping the best balance of “riding the upward trend” and “being cautious about occasional trend reversals”.

For further improving the performance of return maximizing *d*_*L*_ estimation, it is important to examine the effect of CV block size to obtain the optimal *d*_*L*_. Whereas our preliminary test results reveal that the effect from extending the training data period significantly exceeds that of changing the CV block size, it is desirable to thoroughly examine the direct effect of changing CV block size to the return maximizing dL determination.

### 6.4 Number of trades

**Fig 13:**
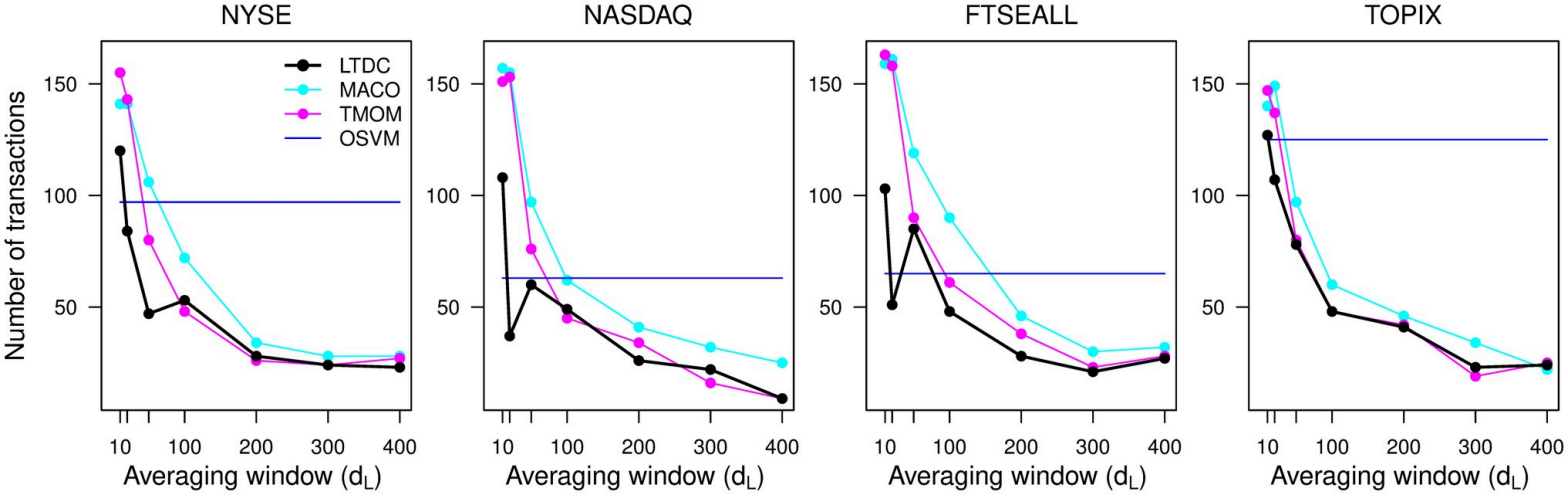
Number of transactions.

The selection of *d*_*L*_ significantly affects the number of trades, and hence, transaction costs. We compute the trading results when the trade is executed only when the buy/sell decision changes from the last month. We depict the relationship between the number of transactions during the 25-year out-of-sample test period and *d*_*L*_ in Fig 13. The number of trades using the trend-following rules decreases as *d*_*L*_ increases because the target trend scale grows as *d*_*L*_ increases, reducing the number of trend reversals. While the number of trades declined significantly in the monthly trading experiment, the cumulative returns corresponding to Fig 13 improved slightly from the results in Fig 5. The trend-following rules’ common optimum *d*_*L*_ during the 25-year test period is not less than 100, and the number of trend-following trades becomes significantly lower around *d*_*L*_ ≥ 100 relative to the non-trend-following OSVM rule. The number of trades using the LTDC is usually the lowest for all *d*_*L*_ values and all indices.

## 7 Discussion

This article studies the problems that trend-following trading confronts, and proposes a simple data-based method of finding an appropriate averaging window which is the key control parameter common for trend-following rules. The limitation of this study is that we have not reached to incorporate our result to provide a practical online trading algorithm. To realize a practical online trading algorithm where traders enjoy the advantage of LTDC while keeping the averaging window at optimal level at every trading decision, it is important to reduce the computational cost of LTDC. The solution key is to design the learning system customized to our specific problem, long-term trend direction classification, to make the simplest possible data-driven system [4–6].

The nature of change we need to handle is determined by our aim, avoiding losses from long-term trend reversals. Hence LTDC requires the longest available training data period to learn the comprehensive relationship between return density shape (via moment statistics) and long-term trend direction including rare large drawdown. Learning longest available data period affects the design of the learning system in two ways: 1) it makes difficult to reduce computational cost of model construction, and 2) it makes the durability of constructed model longer and reduces the gain from re-construction as training period increases.

To reduce the computational cost of windowing techniques in online concept drift learning literature, most approaches aim at reducing the training data such as “forget memory (discard old training data)” or “discard irrelevant training data” [30, 37]. The key for reducing the training cost without shortening training data period, therefore, becomes discriminating the essential information from the training data. Extracting information necessary to learn the cases that are easy to mis-classify seems prominent, but immediate implementation is difficult since it requires further studies on the essential affecting factors to change long-term trend directions.

As to the model durability, there are studies reporting that the classification performance of well pre-trained learning machine based on cross-validation can keep offering better solution than that of online dynamic approach [59, 60]. The SVM of LTDC learns the long-term trend direction in relation with the density shape expressed by return moment statistics, and the extra gain of model re-construction by adding new data decreases as the training data period becomes longer. If the initial learning based on the longest available data has sufficient quality, frequent model re-construction might not be required by implementing some fine-tuning algorithm in generating decision.

Given above discussion, a possible solution is a system combining full-learning model and detection strategy: 1) multiple model (re-)construction with different averaging window (*d*_*L*_) settings by SVM in LTDC framework, and 2) online optimum *d*_*L*_ model selection based on the latest past mean returns. The relationship between return maximizing *d*_*L*_ and the latest past mean returns is estimated at the timing of model (re-)construction. Before every trade decision, the recent return moment statistics is input to the selected model using most appropriate *d*_*L*_ to generate a trade decision. Here, the trend strength (the status of non-stationarity) and trend direction ratio (the degree of class imbalance) is monitored via the recent mean return levels to keep selecting the return-maximizing *d*_*L*_ model.

Another direction to reduce computational cost is employing unsupervised learning which does not require training data. This study adopts supervised learning through k-fold CV since the current unsupervised learning yet remains placed as challenging approach especially in terms of the accuracy [61, 62], and cross-validation-selected algorithm is guaranteed as best-performing algorithm [60]. LTDC rule learns return density shape via moment statistics, therefore, it might also be a prominent direction to pursue unsupervised learning such as online density estimation exploiting more detailed information about the non-stationarity status.

## 8 Conclusions

This study confirms the advantage of trend-following trading in avoiding losses from long-term trend reversals, and empirically demonstrates that the long-term trend direction change (non-stationarity) and trend direction ratio (class imbalance) are the fundamental affecting factors to trend-following trading performance. The difference in the optimum averaging window, *d*_*L*_, by trend direction phase is found to lead to the stability-plasticity dilemma: the trade-off between “keep riding the upward price trend” and “be cautious about sudden trend reversals” in adapting to the time-varying trend reversal risks. That is, the dilemma of selecting a high *d*_*L*_ value for a stable identification of the upward trend direction, and a low *d*_*L*_ value for the sensitivity (plasticity) to capture trend reversal signals. We demonstrate several effective directions to improve conventional trend-following trading performance by outperformance of LTDC rule, and propose a data-based appropriate averaging window finding method adaptive to the market condition.

This study explains that the unstable profitability of trend-following is caused by insufficient chances of avoiding trend reversals. It provides insight to investors that trend-following strategies performs best relative to BHR in risky markets when downside risk protection is most needed. The proposed averaging window finding method is the first data-based guideline easily applicable in practical trading, which is realized through investigation of comprehensive relationship between the optimal averaging window size and various market conditions. It would be more helpful for investors than using some popular averaging window size. Moreover, we are the first to introduce machine classification of smoothed long-term trend direction to generate trading decision, paving the way to explore the significant improvement of long-term downside risk aversion ability.

The remaining problem is incorporating our result to provide the practical online trading algorithm. It is needed to evaluate the practical performance of the suggested learning system combining the full-learning LTDC model and online optimal *d*_*L*_ selection algorithm. Further understanding about long-term trend direction behavior is also the key for discriminating unnecessary instances from the training data to achieve computational cost reduction.

Predicting large long-term trend reversal is quite challenging, and few studies have tackled with it. Trend following is currently the only trading strategy that aims making profit by avoiding large losses from long-term trend reversals. Trend direction classification studies of machine-learning-based approaches have focused on short-term trend direction because of the challenging nature of long-term trend direction classification. By directly handling the essential difficulties lying behind it, non-stationarity with class imbalance, this study helps improve the performance of all trend-following rules, being beneficial to both academia and practitioners.
